# Tracing the Impact of Domestic Storage Conditions on Antioxidant Activity and Lipid Profiles in the Edible Microalgae *Chlorella vulgaris* and *Tetraselmis chui*

**DOI:** 10.3390/md22060254

**Published:** 2024-05-30

**Authors:** Diana Lopes, Felisa Rey, Alexandrina Gomes, Luís Duarte, João Pereira, Marisa Pinho, Tânia Melo, Rosário Domingues

**Affiliations:** 1 Centre for Environmental and Marine Studies (CESAM), Department of Chemistry, Campus Universitário de Santiago, University of Aveiro, 3810-193 Aveiro, Portugal; 2 Mass Spectrometry Centre & Associated Laboratory for Green Chemistry of the Network of Chemistry and Technology (LAQV-REQUIMTE), Department of Chemistry, Campus Universitário de Santiago, University of Aveiro, 3810-193 Aveiro, Portugal

**Keywords:** microalgae, lipidomics, bioactive lipids, PUFA, oxidized lipids, domestic storage

## Abstract

The microalgae *Chlorella vulgaris* and *Tetraselmis chui* are valued for their nutrient-rich content, including lipids and polyunsaturated fatty acids (PUFA). However, little is known about how storage and processing affect their lipid quality. This study aimed to assess the impact of domestic storage and cooking practices in dried biomass of *C. vulgaris* and *T. chui*. Four conditions were tested: control (newly opened package), light (storage at room temperature and daily light regimen for three weeks), frozen (storage in the freezer at −20 °C for three weeks), and heated (three cycles of 90 min at 100 °C). Lipid extracts were analyzed by GC-MS and LC-MS, and antioxidant activity through DPPH and ABTS radical scavenging assays. Tested storage conditions promoted a decrease in fatty acid content and in diacyl/lyso lipid species ratios of phospholipid (PC/LPC, PE/LPE) and betaine lipids (DGTS/MGTS). Lipid extracts from light treatment showed the lowest antioxidant activity in *C. vulgaris* (ABTS, IC40: 104.9; DPPH, IC20: 187.9 ± 15.0), while heat affected the antioxidant activity of *T. chui* (ABTS, IC40: 88.5 ± 2.8; DPPH, IC20 209.4 ± 10.9). These findings underscore the impact of managing storage and processing conditions to optimize the nutritional and functional benefits of *C. vulgaris* and *T. chui* in food and feed applications.

## 1. Introduction

Current consumer demand for natural-origin and functional food is increasing the research of matrices with health-promoting properties. Within this realm, microalgae have emerged as prominent candidates, boasting a diverse array of high-value nutritional molecules and demonstrating potential as feedstock for the production of bioactive compounds [1,2,3,4]. 

*Chlorella vulgaris* and *Tetraselmis chui* from the Chlorophyta phylum have garnered significant attention for their incorporation into functional food and feed formulations [5]. *Chlorella vulgaris* is a freshwater green microalga approved for human nutrition, esteemed for its high protein content, and a source of nutritional lipids [6]. This microalga has high photosynthetic ability and adaptability to various growth conditions, including autotrophic, mixotrophic, and heterotrophic modes, making it well-suited for large-scale cultivation and commercial production [7]. On the other hand, *T. chui* is a flagellated marine green microalga boasting rapid growth, a non-toxic nature, and remarkable nutritional value composed mainly of proteins with all essential amino acids, carbohydrates, and fats [1,8]. Due to its antioxidant properties and nutritional significance, it is extensive used as efficient feed in the aquaculture industry [9,10,11]. Moreover, dried biomass of *T. chui* has been authorized in the EU as novel food and food supplement (Commission Implementing Regulation (EU) 2017/2470) [12,13,14].

The significance of these microalgae species lies in their potential as sources of beneficial compounds, particularly lipids and polyunsaturated fatty acids (PUFA), including essential omega-3 (*n*-3) and omega-6 (*n*-6) PUFA-like alpha-linolenic acid (18:3 *n*-3, ALA) and linoleic acid (18:2 *n*-6, LA), respectively. Given that ALA and LA are not synthesized in mammalian cells, their intake through dietary sources is crucial for the proper metabolism of these organisms [15]. Furthermore, in vitro assays have demonstrated that lipid extracts of *C. vulgaris* exhibits antioxidant and anti-inflammatory properties [16,17], underscoring the functional significance of these compounds. 

However, the biochemical profile of food matrices is significantly influenced by storage conditions [18,19]. Regarding lipids, during the food manufacturing and storage, they are susceptible to degradation when exposed to excess humidity, heat, and light. These factors can prompt the hydrolysis of esters bonds, leading to the release of fatty acids (FA) chains and formation of lyso forms (e.g., lysophosphatidylcholines) [20,21]. Furthermore, lipids are prone to oxidation, which can compromise both sensory attributes and health-promoting qualities, resulting in the production of low molecular weight off-flavor compounds, depletion of antioxidants, and accumulation of free radicals [22]. The stability of lipids against oxidation is primarily influenced by oxygen, temperature, and light [21]. Moreover, household practices like freezing and cooking can also tiger lipid oxidation, leading in loss of quality [23]. 

In this regard, assessing the stability of the biochemical composition of algae biomass under various storage scenarios becomes important. Despite the promising attributes of microalgae-derived lipids, their vulnerability to degradation poses a critical challenge to maintaining the integrity and efficacy of the biomass [24]. Storage conditions play a pivotal role in preserving the pigment, FA, and lipid profiles, as well as antioxidant activities inherent to microalgae lipid extracts. Understanding the changes induced by different conditions is crucial for optimizing strategies to preserve microalgae biomass, ensuring the high-quality lipid extracts for commercial applications. 

This study aims to evaluate the impact of simulated domestic storage and cooking practices of dried biomass of *C. vulgaris* and *T. chui* on their lipid extract, focusing on alteration in the lipid composition and antioxidant activity. Four conditions were selected for analysis: condition 1—control: biomass stored at room temperature under vacuum, protected from light and opened for the first time on the day of analysis; condition 2—light: biomass stored at room temperature in an open container exposed to light and oxygen for 3 weeks; condition 3—frozen: biomass stored in the freezer at −20 °C for three weeks; and condition 4—heated: biomass heated at 100 °C for 90 min during three cycles. Lipids were extracted from the biomass subject to different conditions, and their FA and lipid profiles were analyzed using gas chromatography–mass spectrometry (GC-MS) and liquid chromatography (LC-MS), respectively. Additionally, the antioxidant activity of the lipid extracts was evaluated by 2,20-Azino-bis-3-Ethylbenzothiazoline-6-Sulfonic Acid (ABTS) and 2-Diphenyl-1-Picrylhydrazyl (DPPH) radical scavenging assays.

## 2. Results

### 2.1. Fatty Acids Profile and Lipid Quality Indices

The lipid yield of *C. vulgaris* under control, light, frozen, and heated conditions corresponded to 4.6 ± 0.7%, 5.7 ± 0.8%, 5.0 ± 0.3%, and 6.6 ± 0.6% of dry weight (DW), respectively. For *T. chui*, this yield corresponds to a 3.9 ± 0.8%, 5.7 ± 2.0%, 6.3 ± 0.4%, and 6.5 ± 0.9% DW, respectively.

The FA profile of *C. vulgaris* and *T. chui* exposed to distinct storage and analyzed by gas chromatography–mass spectrometry (GC-MS) allowed for identifying a total of eight FA in *C. vulgaris* (Table 1) and eleven in *T. chui* (Table 2). *Chlorella vulgaris* profile showed the FA 16:0, linoleic acid (LA, 18:2*n*-6), and the alpha linolenic acid (ALA, 18:3*n*-3) as the most abundant ones (Table 1). In the *T. chui* profile, 16:0, 16:4*n*-3, and ALA were the predominant FA (Table 2). 

In *C. vulgaris*, the FA analysis revealed a decrease of each fatty acid across all conditions compared to the control condition, which was most pronounced under light and frozen conditions. Specifically, the highest reduction in light condition was observed in the most unsaturated FA, such as 16:3*n*-3 and 18:3*n*-3, which exhibited significant differences compared to the other conditions (Table 1). Additionally, the *n*-6/*n*-3 ratio remained lower than 1 across all conditions, with PUFA exhibiting the lowest value under light condition.

Lipid quality indices, atherogenicity index (AI) and thrombogenicity index (TI), showed no differences in all conditions (AI and TI equal to 0.2 ± 0.0), with exception of ice conditions (TI equal to 0.1 ± 0.0). The hypo/hypercholesterolemic ratio (h/H) was lower in control and frozen conditions but significative differences were not recorded between storages conditions (Table 1).

In the FA profiles of *T. chui*, the most pronounced differences were observed in the unsaturated FA 16:4*n*-3 and 18:3*n*-3, which exhibited reductions under light conditions. However, no significant differences were recorded in FA composition between storage conditions (Table 2). Similarly to *C. vulgaris*, *T. chui* also exhibited a consistently low *n*-6/*n*-3 ratio (0.2) across all conditions, with *n*-3 PUFA displaying the lowest value under light conditions. Lipid quality indices showed no significative differences between conditions. The AI was equal to 0.3 ± 0.0 with exception of control conditions (0.4 ± 0.0), while the TI was equal to 0.2 ± 0.0 in control and light conditions and lower in frozen and heated conditions (0.1 ± 0.0). The hypo/hypercholesterolemic ratio was lower in control condition although with no significative differences.

### 2.2. Lipid Ratios

**Figure 1:**
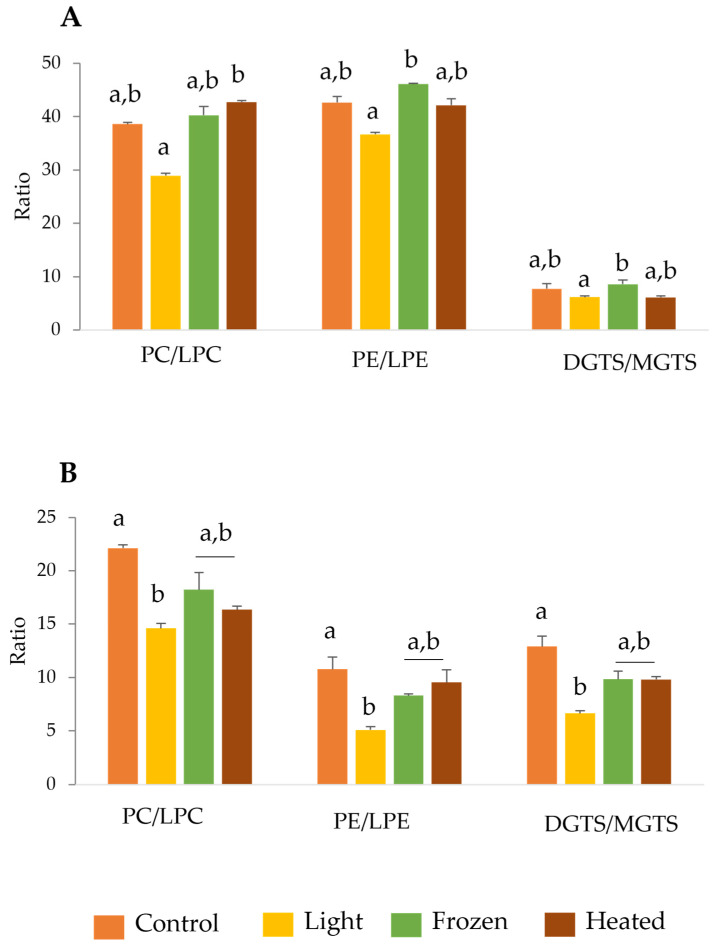
Polar lipid ratios between diacyl and lyso forms of phosphatidylcholine (PC) and lyso-phosphatidylcholine (LPC); phosphatidylethanolamine (PE) and lyso-phosphatidylethanolamine (LPE); diacylglyceryltrimethylhomoserine (DGTS) and monoacylglyceryltrimethylhomoserine (MGTS) for *Chlorella vulgaris* (**A**) and *Tetraselmis chui* (**B**) exposed to different storages conditions (control, light, frozen, and heated). Values are means of the normalized extracted ion chromatograms (XIC) areas of three replicates (*n* = 3) ± standard deviation. Different letters represent significant differences among conditions (q < 0.05, Kruskal–Wallis test followed by Dunn’s post-hoc comparisons).

The analysis of lipidomics data using the MS Dial software allowed for the identification of lyso species of phospholipids phosphatidylcholine (PC) and phosphatidylethanolamine (PE) and betaine lipids diacylglyceryltrimethylhomoserine (DGTS). Our analysis was focused on semi-quantities of both diacyl (PC, PE, DGTS) and the lyso forms lysophosphatidylcholine, lysophosphatidylethanolamine, and monoacylglyceryltrimethylhomoserine (LPC, LPE, and MGTS). Subsequently, the following ratios were calculated: PC/LPC, PE/LPE, and DGTS/MGTS, for both *C. vulgaris* and *T. chui* (Figure 1). 

The analysis of lipid ratios between the different storage conditions revealed that the light condition exhibited the lowest ratios across the different lipid groups in both microalgae species, *C. vulgaris* and *T. chui*. These results revealed an increment in the abundance of lyso lipid species in the light condition compared to the other conditions in both phospholipids and betaine lipids. In *C. vulgaris*, the PC/LPC ratio was significant different between light and heated treatment, while PE/LPE e DGTS/MGTS ratios showed significant differences between light and frozen treatments (Figure 1A). In *T. chui*, the three calculated ratios were significant differences between control and light treatments (Figure 1B).

Interestingly, lipid species of oxidized phospholipids were only identified in *C. vulgaris*. Six lipid species of oxidized PC and four oxidized PE were identified, and the non-oxidized/oxidized species ratio was calculated (Table 3). The ratio corresponding to light-exposed samples exhibited lower values, indicating an increment of oxidized species in this treatment. 

**Figure 2:**
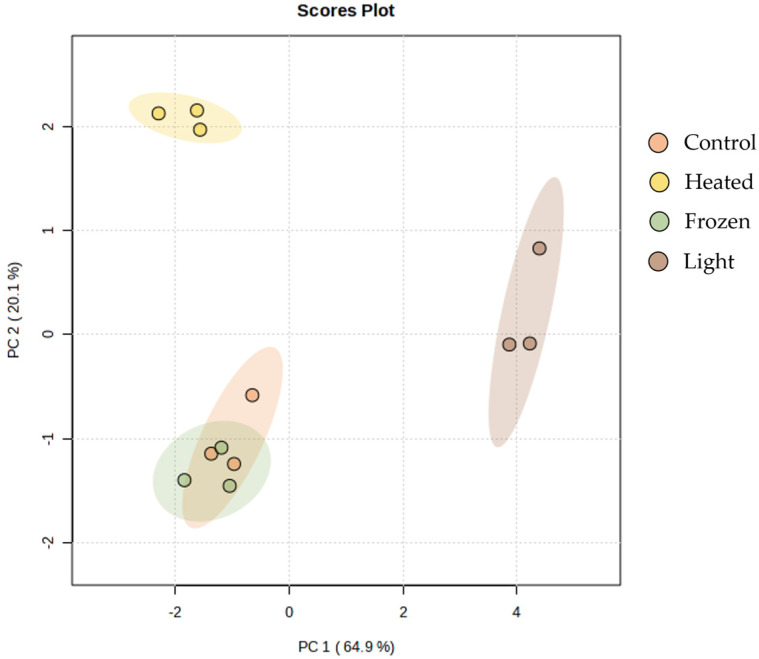
Principal component analysis (PCA) of log-transformed normalized extracted-ion chromatogram (XIC) areas of non-oxidized and oxidized phosphatidilcholines and phosphatidylethanolamines lipid species identified in *Chlorella vulgaris* exposed to different conditions (control, light, frozen, and heated). Three (*n* = 3) samples by condition were analyzed.

The PCA calculated using the normalized extracted ion chromatogram (XIC) areas of non-oxidized and oxidized lipids species showed that its two principal components explained 85% of total variance, with principal component 1 (PC 1) and principal component 2 (PC 2) axes explaining 64.9% and 20.1%, respectively (Figure 2). A clear discrimination can be observed between samples from different storage conditions; while control and frozen samples overlapped, light-exposed samples were separated from this group along PC 1, and heated-exposed samples along PC 2 (Figure 2).

### 2.3. Antioxidant Activity

The *in chemico* antioxidant activity of lipid extracts of *C. vulgaris* and *T. chui* exposed to different conditions was determined by 2,20-Azino-bis-3-Ethylbenzothiazoline-6-Sulfonic Acid (ABTS) and 2-Diphenyl-1-Picrylhydrazyl (DPPH) scavenging assays. The concentration of lipid extract required to achieve 40% inhibition of ABTS radicals (IC40) and 20% inhibition of DPPH radicals (IC20) was determined.

An IC40 of 32 ± 11.0 μg mL^−1^ and IC20 of 97.8 ± 27.2 μg mL^−1^ were obtained in ABTS and DPPH assays, respectively, for *C. vulgaris* under control conditions (Table 4). Remarkably, in both assays, the control condition showcased the highest antioxidant activity, while the light condition demonstrated the lowest antioxidant activity.

Regarding the antioxidant activity of lipid extracts of *T. chui* exposed to different storage conditions (Table 5), the results of control condition showed different tendencies in ABTS and DPPH assays. In ABTS assay, the best antioxidant activity was achieved in the frozen condition, with an IC40 of 29 ± 5.4 μg mL^−1^, whereas the lowest activity was obtained for heated samples with an IC40 of 88.5 ± 2.8 μg mL^−1^. On the other hand, in the DPPH assay, the control condition exhibited the best antioxidant activity with an IC20 of 70.6 ± 23.1 μg mL^−1^, while the heated condition again displayed the lowest antioxidant activity with an IC20 of 209.4 ± 10.9 μg mL^−1^.

## 3. Discussion

The microalgae species *C. vulgaris* and *T. chui* are highly valued in functional food and feed applications, due to their abundance in beneficial lipids, particularly omega-3 PUFA and their documented bioactive lipid species, as well as other intriguing compounds [9,17]. 

In this study a slight increase in lipid yield was verified under heated conditions for both *C. vulgaris* and *T. chui*. This phenomenon could be attributed to protein denaturation at higher temperatures, which enhances the accessibility of extraction solvents to membranes [25]. Additionally, the disruption of membrane structures induced by both low (frozen condition) and high (heated condition) temperatures may enhance lipid accessibility, thereby facilitating their extraction. Interestingly, a similar increase in lipid extractability was observed in *T. chui* stored on freezer. This phenomenon of increased lipid extractability with different temperature has been previously reported for other food matrices [26].

The FA profiles of both microalgae were analyzed using basic catalysis transmethylation, focusing on esterified FA, which are of particular interest in this study due to their nutritional relevance in food and feed applications. It is important to note that this method does not methylate free fatty acids (FFA), and therefore these FFA were not included in the FA analysis. The results are consistent with previous studies, with 16:0, linoleic acid (LA, 18:2*n*-6), and alpha-linolenic acid (ALA, 18:3*n*-3) being the most abundant in the lipid extracts of *C. vulgaris* [17,27], and 16:0, 16:4*n*-3, and ALA being predominant for *T. chui* [28,29]. Linoleic acid and ALA are essential *n*-6 and *n*-3 FA, respectively, crucial for human health as they cannot be synthesized by the body and must be obtained through diet. They serve as precursors of long-chain essential PUFA such as arachidonic acid (AA, 20:4*n*-6), eicosapentaenoic acid (EPA, 20:5*n*-3), and docosahexaenoic acid (22:6*n*-3), which play a relevant role in cognitive and immune functions. The FA profile of *C. vulgaris* and *T. chui*, although without significant differences, showed a decrease in PUFA in light conditions compared to control conditions. This observation aligns with the known susceptibility of PUFA to chemical modifications, such as autoxidation or metal-catalyzed oxidation, attributed to their multiple double bonds that render them vulnerable to electrophilic attack [30]. Moreover, both frozen and heated conditions also led to a decrease in PUFA in *C. vulgaris*, although this effect was less pronounced than under light conditions. Interestingly, the SFA of both *C. vulgaris* and *T. chui* also showed a decrease under light, frozen, and heated conditions compared to the control condition. This reduction was already observed in other food matrices during storage [31]. The decrease in SFA as well as other FA could potentially be linked to the action of lipases. Lipases are enzymes widely distributed in nature, exhibiting high specificity and selectivity for their substrates. They hydrolyze lipids, releasing free fatty acids, and demonstrate selectivity towards specific FA at particular regions bonded to specific carbons of glycerol. They are considered stable and versatile, able to catalyze reactions under extremes of temperature, pH, and organic solvents [32,33]. Given that the analyzed FA in this work reflects esterified FA, the observed decrease in FA under different storage conditions could indeed be associated with membrane damage, allowing lipases greater access to lipids and subsequently reducing esterified FA levels. The *n*-6/*n*-3 ratio for a healthy diet should be 6:1 [34,35]. Reducing the intake of *n*-6/*n*-3 ratio can enhance blood lipids and quality of life, and is crucial for preventing cardiovascular disease [36]. The lack of differences in the *n*-6/*n*-3 ratio between treatments suggests that the loss of FA was proportional in PUFA regardless of the double bond position. Although the different conditions tested, the amount of *n*-3 PUFA was always higher than *n*-6 PUFA, resulting in consistently low *n*-6/*n*-3 ratio across all conditions for both *C. vulgaris* and *T. chui*. This result indicates that despite the exposed conditions, these microalgae maintain a nutritionally beneficial ratio. The AI and TI indices assess the potential for stimulating platelet aggregation, as well as the development of blood clots, atheroma, and thrombus formation [37,38]. Consequently, smaller IA and IT values suggest greater protective potential against cardiovascular diseases [39]. This study revealed that these indices were not significantly affected by the tested storage conditions, with both *C. vulgaris* and *T. chui* exhibiting very similar values (lower than 1 in both species). The indices observed in *C. vulgaris* and *T. chui* are consistent with those previous reported [17,40]. The h/H index evaluates the ratio between hypocholesterolemic and hypercholesterolemic FA, reflecting their association with cholesterol metabolism. High h/H values are considered more favorable for human health [41,42]. In this context, *C. vulgaris* displayed higher h/H indices compared to *T. chui*, without significative differences between storage conditions for both microalgae. The h/H values of both *C. vulgaris* and *T. chui* were higher than those of fish lipids (ranging from 0.6 to 2.5) [34,42,43], with fish traditionally considered a source of healthy lipids. This highlights these microalgae as a promising alternative source of healthy lipids.

The detailed lipid characterization of *C. vulgaris* and *Tetraselmis* sp. were already reported in previous studies [16,44,45]. Therefore, this study aimed to elucidate lipid alterations under different storage conditions by identifying lipid species indicative of degradation, particularly lyso forms and oxidized species. The analysis revealed a higher abundance of lyso species in the PC, PE, and DGTS classes. Consequently, this study was focused on these lipid classes, investigating the PC/LPC, PE/LPE, and DGTS/MGTS ratios to gain insights into lipid degradation processes. Curiously, for *T. chui*, both frozen and heated conditions affected the PE and DGTS classes in a very similar manner. The authors speculate that these differences may be attributed to lipid localization within cellular membranes. Given that phospholipids and betaine lipids constitute extraplastidial membranes, with potential variations between inner and outer membranes, lipids more impacted in *C. vulgaris* and *T. chui* could potentially reside in the outer membrane, rendering them more susceptible to modifications. These findings highlight the potential impact of varying storage conditions on lipid composition, which appears to be species-dependent among microalgae. Previous studies investigating the storage of bivalve mollusks have revealed that exposure to low temperatures result in an increase in lyso phospholipids, including LPC and LPE [46,47]. This increase serves as a marker of prolonged refrigeration. Interestingly, a similar elevation in lyso phospholipids is observed when bivalves are stored in non-refrigerated seawater with temperatures exceeding 25 °C.

In *C. vulgaris*, oxidized lipid species were identified in PC and PE classes, with the non-oxidized/oxidized ratio consistently lower in samples exposed to light storage conditions. Conversely, in other storage conditions, this ratio closely resembled the control ratio. These results suggest that these species might naturally occur in the biomass of *C. vulgaris*; however, they experience an increase under prolonged exposure to natural light, which was particularly high in PE36:1;O species. PCA analysis reveals that the control and frozen conditions exhibit closer proximity than the light and heated conditions, suggesting distinct oxidation patterns influenced by the specific environmental conditions they experienced. Lipid peroxidation involves the attack of oxidants, such as free radicals or nonradical species, on lipids containing carbon–carbon double bonds, particularly PUFA. This process results in the abstraction of hydrogen from a carbon atom, followed by oxygen insertion, leading to the formation of lipid peroxyl radicals and hydroperoxides [48,49]. Phospholipids are common targets of peroxidative modification. Additionally, lipids can be oxidized by enzymes like lipoxygenases that are quite active in plants and algae [50]. The oxidation of lipids can give rise to harmful compounds and thus being detrimental to human health [51]. Intriguingly, in *T. chui* were not detected oxidized PC and PE species, suggesting that the microalga’s cell structure integrity may offer enhanced protection against lipid oxidation during storage conditions or that they are present in a lower concentration than in *C. vulgaris*.

In terms of antioxidant activity, in *C. vulgaris*, both the ABTS and DPPH assays consistently demonstrate lower antioxidant activity in the lipid extracts from light-exposed samples. The values of the control condition of *C. vulgaris* align with previously reported results for *C. vulgaris* cultured under autotrophic conditions [16,17]. However, in *T. chui*, lipid extract from heated-exposed samples displayed the lowest antioxidant activity. This variance in antioxidant activity may be linked to PUFA, suggesting that the reduction observed in light-exposed *C. vulgaris* samples could stem from higher degradation of these FA. However, this phenomenon was not observed in *T. chui*. It is worth noting that besides lipids, other compounds such as photosynthetic pigments and lipid-soluble compounds (e.g., phenolic compounds) also contribute significantly to antioxidant activity. These compounds could potentially have a greater influence on the decreased antioxidant activity observed in heat-exposed *T. chui*. 

## 4. Materials and Methods

### 4.1. Microalgae Samples

The *C. vulgaris* and *T. chui* biomasses were provided by Allmicroalgae Natural Products S.A. (Pataias, Portugal). The species were grown autotrophically using Guillard’s F2 culture medium adapted to local water. *T. chui* was supplemented with a magnesium mixture (Necton, Olhão, Portugal) and NaCl (Salexpor, Coimbra, Portugal) at a salinity of 30 g·L^−1^. The microalgae cultures were cultivated in 5 L flask reactors under continuous 700 μmol photons·m^2^ s^−1^ light exposure for 7 to 15 days. Five 5 L flask reactors were utilized to inoculate one 0.1 m^3^ outdoor flat panel (FP) reactor, which was subsequently scaled up to 1 m^3^ FPs. Of the five FPs, four were used as inoculum for a 10 m^3^ tubular photobioreactor (PBR). The PBR was exposed to ambient light and temperature conditions until reaching the stationary phase. A sprinkler-like irrigation system was employed to maintain the temperature of the PBR below the maximum limit, while pulse injections of CO_2_ were utilized to stabilize the pH. Temperature and pH conditions in the 10 m^3^ PBRs were operated as described in [40].

The biomass was recovered through centrifugation and subsequently dried. Microalgae at approximately 50 g·L^−1^ were dried by atomization in a spray dryer with an evaporation capacity of 150 kg water·h^−1^. Drying was rapidly achieved using an air stream at 215 ± 5 °C, with the outlet air temperature with the biomass powder measured at 92 ± 3 °C. The resulting powder was collected via a cyclone and stored under protection from light and humidity.

### 4.2. Evaluated Conditions

The *C. vulgaris* and *T. chuii* biomass was analyzed from sealed commercial packages stored at room temperature under vacuum, protected from light, and opened for the first time on the day of analysis, serving as the baseline control condition (condition 1—control). Alongside, biomass was separated from the same packages to explore three additional conditions: condition 2—light; condition 3—frozen; and condition 4—heated. For the second condition, the biomass was stored at room temperature in an open container exposed to light and oxygen for 3 weeks (condition 2—light). For the third condition, the biomass was stored involved in a freezer at −20 °C for 3 weeks (condition 3—frozen), while the fourth condition the biomass was heated at 100 °C during 270 min—three cycles of 90 min (condition 4—heated).

### 4.3. Lipid Extraction

Folch’s method [52] was used to extract total lipid content of *C. vulgaris* and *T. chui* (Allmicroalgae Natural Products S.A) samples under the four different experimental conditions. The extraction was executed individually for each condition and three replicates were made. A volume of 2 mL of DCM:MeOH (2:1, *v*/*v*) was added to 25 mg of microalga powder in glass tube and vortexed for 2 min, followed by incubation on ice for 30 min in an orbital shaker (Stuart SSL2 reciprocating shaker). Afterwards, the mixture was centrifuged at 2000 rpm for 10 min (Centurion Scientific limited prO-analytical centrifuge). This step was repeated three more times for each replicate. The organic phase was completely dried under a nitrogen stream. To remove non-lipid compounds, the nitrogen-dried extract was dissolved in 3 mL DCM:MeOH (2:1, *v*/*v*), and 0.75 mL of Milli-Q water was added. The mixture was vortexed for 2 min followed by centrifugation at 2000 rpm for 10 min to induce phase separation. This was followed by collection of the organic phase (lower phase), and re-extraction of the aqueous phase by addition of 2 mL of DCM, then vortexed for 1 min and centrifuged for 5 min at 2000 rpm (two more times). The combined organic phases were filtered to a new glass tube using a paper filter (Whatman^TM^, Kent, UK) to remove eventual suspended particles and dried under nitrogen stream. The lipid extracts were then transferred to amber vials and stored at −20 °C until further analysis.

### 4.4. Analysis of Esterified Fatty Acid Analysis by Gas Chromatography–Mass Spectrometry

Fatty acid methyl esters (FAME) were prepared from the by base-catalyzed transmethylation of the *C. vulgaris* and *T. chui* lipid extracts. The dried lipid extracts were mixed with 1 mL of an internal standard solution (1.30 µg mL^−1^ of methyl nonadecanoate in *n*-hexane) followed by addition of 200 μL of KOH solution (2.0 M in MeOH) and vortexed for 2 min. Afterwards, 2 mL of saturated NaCl solution (10 mg mL^−1^ in water) was added, followed by centrifugation at 2000 rpm for 5 min. The organic phase (600 µg of upper phase) was collected into a new glass tube and dried under a nitrogen gas steam. To prepare the sample for GC-MS analysis, the dried FAMEs were dissolved in 100 μL of 99% *n*-hexane and 2 µL of this solution were injected in a GC-MS (Agilent Technologies 6890N Network, Santa Clara, CA, USA), equipped with a DB-1 column 30 m in length, 0.25 mm internal diameter, and 0.1 m film thickness (J&W Scientific, Folsom, CA, USA). The GC-MS system was coupled with an Agilent 5973 Network Mass Selective Detector (Agilent Technologies) operating with an electron impact mode at 70 eV and scanning a range of 40–500 *m*/*z* in a 1 s cycle in full-scan mode acquisition.

The temperature of the oven was programmed to start at 58 °C (hold time 2 min), followed by a linear increase to 160 °C at a rate of 25 °C per min, then a linear increase of 2 °C per min to 210 °C, and finally a 20 °C per min increase until reaching 225 °C (hold time 15 min). The injector and detector temperatures were set at 220 °C and 230 °C, respectively. Helium gas served as the carrier at flow rate of 1.7 mL min^−1^. Identification of the FAMEs was conducted by comparing the retention time and mass spectrum of each FAME with a mix of FAME standards (C6–C24, Supelco 37 Component FAMEs Mix). Confirmation was achieved through comparison with the Wiley chemical database and the spectral library, the “NIST Lipid Library”. Calculation of FA amounts was carried out following peak integration and normalization using the amount of internal standard methyl nonadecanoate.

The lipid quality indices, namely, the atherogenicity (AI) and thrombogenicity (TI) indices as well as the hypocholesterolemic/hypercholesterolemic (h/H) ratio, were obtained from the formulas described below [53]:

$$AI=\frac{12:0+4\times 14:0+16:0}{\sum MUFA+\sum PUFA\;n-6+\sum PUFA\;n-3}$$

$$TI=\frac{14:0+16:0+18:0}{(0.5\times \sum MUFA)+(0.5\times \sum PUFA\;n-6)+(3\times \sum PUFA\;n-3)+\frac{\sum PUFA\;n-3}{\sum PUFA\;n-6}}$$

$$h/H=\frac{cis\;18:1+\sum PUFA}{12:0+14:0+16:0}$$

### 4.5. Analysis of Lipid by C18-MS

The lipidome analysis was conducted via liquid chromatography C18, utilizing an Ascentis Express 90 Å C18 HPLC column (15 cm × 2.1 mm; 2.7 μm, Supelco, Sigma-Aldrich, Bellefonte, PA, USA), integrated into an HPLC system (Ultimate 3000 Dionex, Thermo Fisher Scientific, Bremen, Germany) with an autosampler linked to the Q-Exactive^®^ hybrid quadrupole Orbitrap mass spectrometer (Thermo Fisher, Scientific, Bremen, Germany). A volume of 5 μL of each sample, equivalent to 10 μg of lipid extract (in 10 µL of dichloromethane), 82 µL of a solvent system comprising 50% isopropanol/50% methanol, and 8 µL of phospholipid standards mixture was injected into the HPLC column at a flow rate of 260 μL/min. The column oven temperature was maintained at 50 °C. A 33 min gradient was employed, starting with 32% mobile phase B at 0 min, 45% B at 1.5 min, 52% B at 4 min, 58% B at 5 min, 66% B at 8 min, 70% B at 11 min, 85% B at 14 min, 97% B at 18 min, 97% B at 25 min, 32% B at 25.01 min, and 32% B at 33 min. Mass spectrometry was conducted in positive and negative ionization modes over an m/z range of 200–2000 with a resolution of 70,000. Tandem MS (MS/MS) experiments were carried out using a top 10 data-dependent acquisition method, with the most abundant precursor ions selected for fragmentation via higher energy dissociation (HCD). The MS/MS spectra were obtained with a resolution of 17,500, and lipid species were identified using mass spectrometry–data-independent analysis (MS DIAL) v4.70 software.

PC, LPC, PE, LPE, DGTS, and MGTS lipid species were identified in positive ionization modes using the raw files acquired in MS/MS mode and converted by the ABF converter against the lipid database provided by MS-DIAL 4 software. Tolerances for MS and MS/MS search were set at 0.01 Da and 0.05 Da, respectively. Following MS-DIAL identifications, peaks were automatically integrated in MZmine 3.9.0 for LC–MS data normalization. Integrated peak areas of extracted ion chromatograms (XIC) were exported from MZmine 3.9.0 software, and each specie’ peak area was normalized against the peak area of the lipid standard with the closest retention time. The resulting matrix (.xls) with normalized areas of all species facilitated the study of origin-driven variations at the lipid species level. 

### 4.6. Evaluation of Antioxidant Activity of Lipid Extracts by DPPH and ABTS Radical Scavenging Activity

The antioxidant activity of *C. vulgaris* and *T. chui* lipid extracts was evaluated by measuring its capacity to scavenge DPPH [54] and ABTS radicals [55]. For the DPPH assay, a stock solution of DPPH^●^ in ethanol (250 µmol L^−1^) was prepared and diluted to prepare a solution with an absorbance value of approximately 0.9, measured at 517 nm using a UV–vis spectrophotometer (Multiskan GO 1.00.38, Thermo Scientific, Hudson, NH, USA). To evaluate the lipid extract scavenging potential, a volume of 150 µL of C. vulgaris and *T. chui* lipid extracts (12.5, 62.5, 125, and 250 µg mL^−1^ in ethanol) and 150 µL of Trolox standard solution (5, 12.5, 25, and 37.5 µmol L^−1^ in ethanol) were placed in each well followed by addition of 150 µL of DPPH^●^-diluted solution, followed by an incubation period of 120 min, with absorbance measured at 517 nm every 5 min [56]. 

For ABTS assay, a solution of ABTS (3.5 mmol L^−1^) was prepared by combining 10 mL of ABTS stock solution (7 mmol L^−1^ in water) with 10 mL of potassium persulfate, K_2_S_2_O_8_ (2.45 mmol L^−1^ in water). This mixture was left for 12 h at room temperature and then diluted in ethanol to obtain an absorbance value of ~0.9 measured at 734 nm using a UV–vis spectrophotometer (Multiskan GO 1.00.38, Thermo Scientific, Hudson, NH, USA). To assess the scavenging potential of extracts, 150 µL of *C. vulgaris* and *T. chui* lipid extracts (12.5, 62.5, 125, and 250 µg mL^−1^ in ethanol) or 150 µL of Trolox standard solution (5, 12.5, 25, and 37.5 µmol L^−1^ in ethanol) were added to each well, followed by the addition of 150 µL of ABTS^•+^ diluted solution, and incubated for 120 min, with absorbance measurements at 734 nm every 5 min [57]. Radical reduction was monitored by measuring the decrease in absorbance during the reaction, thereby quantifying radical scavenging, which is accompanied by a radical color change.

The free radical scavenging activity of samples was determined as the percentage of inhibition of DPPH and ABTS radicals according to Equation (1).

(1) $$Inhibition\left(\%\right)=\frac{Abs\;radical-\left(Abs\;Sample-Abs\;Control\right)}{Abs\;radical}\times 100$$

The concentration of samples capable of reducing 20% and 40% of DPPH and ABTS radical, respectively, after 120 min (IC20 and IC40, respectively) were calculated by linear regression using the concentration of samples and the percentage of the inhibition curve. The antioxidant activity was also expressed as Trolox equivalents (TE, µmol Trolox.g^−1^ of lipid extract), according to Equation (2)

(2) $$TE(\mu mol\;Trolox.g^{-1}\;of\;lipid\;extract)=\frac{IC\left(20\;or\;40\right)\;Trolox\;(\mu mol\;g^{-1})}{IC\left(20\;or\;40\right)\;Sample\;(\mu g\;{mL}^{-1})}\times 1000$$

### 4.7. Statistical Analysis

The non-parametric Kruskal–Wallis test was used to identify significant differences between microalga storage conditions in fatty acid content and PC/LPC, PE/LPE, and DGTS/MGTS ratios. Post-hoc comparisons were performed using Dunn’s tests. *p*-values were corrected for multiple testing using the Benjamin–Hochberg method (q values). Differences with q-value < 0.05 were considered statistically significant. These statistical analyses were performed using R version 4.1.0 (R Core Team, 2021) in rStudio 2023.6.1.524 (Posit Team 2023). Non-oxidized and oxidized ratios of phosphatidilcholine and phosphatidylethanolamine lipid species identified in *Chlorella vulgaris* (Figure S1) were calculated using the normalized extracted ion chromatograms (XIC). Ratio data were log-transformed and auto-scaled and principal component analysis (PCA) was performed to assess the clustering of the four storage treatments in Metaboanalyst 6.0. software.

## 5. Conclusions

The utilization of functional ingredients to achieve beneficial effects through diet is currently expanding in human and animal feeding. However, storage conditions can significantly impact the molecular composition of food ingredients, namely, FA and lipid profiles. 

This study demonstrated a decrease in FA content across all tested conditions, with the most pronounced reduction in PUFA under light exposure treatment. Among the microalgae species studied, *C. vulgaris* exhibited the highest susceptibility to this reduction. The alterations in diacyl species/lyso species ratio of phospholipid (PC and PE) and betaine lipids (DGTS) suggest a degradation in the quality of these lipids with the treatments. Additionally, this study also demonstrated how exposition to light and heat reduce the antioxidant potential of both *C. vulgaris* and *T. chui*. These results emphasize the need for the proper preservation of microalgae biomass to realize their full potential in nutritional and functional food and feed applications. Future research should focus on evaluating the stability of biomass under different preservation methods to optimize storage and conservation techniques, ensuring the maintenance of biomass quality. Such advancements are crucial for the efficient use of microalgae in various applications.

## Figures and Tables

**Table 1:** Fatty acid profiles and lipid quality indices of *Chlorella vulgaris* exposed to different storages conditions (control, light, frozen, and heated), determined by gas chromatography–mass spectrometry (GC–MS) and expressed in μg mg^−1^ of lipid extract. Values are presented as average of three replicates (*n* = 3) ± standard deviation. The fatty acids 16:1 and 18:1 correspond to the sum of two peaks for which the exact position of unsaturation within the chain have not been clearly assigned. Different letters in the same line represent significant differences among conditions (q < 0.05, Kruskal–Wallis test followed by Dunn’s post-hoc comparisons).

Fatty Acids	Control	Light	Frozen	Heated
16:0	52.3 ± 4.8	35.1 ± 6.2	37.5 ± 2.9	46.2 ± 3.2
16:1	9.3 ± 0.7	6.2 ± 1.1	7.3 ± 0.9	9.2 ± 0.7
16:2*n*-6	21.4 ± 1.5	14.2 ± 2.4	16.9 ± 2.3	20.8 ± 1.2
16:3*n*-3	38.7 ± 2.7 ^a^	24.1 ± 4.0 ^b^	30.8 ± 4.7 ^a,b^	35.2 ± 2.0 ^a,b^
18:0	13.4 ± 5.3	8.4 ± 1.6	6.3 ±0.8	10.6 ± 0.4
18:1	26.9 ± 1.7	19.3 ± 3.0	21.2 ± 1.8	28.0 ± 2.3
18:2*n*-6	61.9 ± 3.5	46.3 ± 7.3	49.9 ± 5.8	62.7 ± 4.7
18:3*n*-3	72.3 ± 4.2 ^a^	45.9 ± 7.3 ^b^	56.7 ± 7.4 ^a,b^	65.5 ± 4.1 ^a,b^
*n*-3	111.0 ± 6.9	70.0 ± 11.2	87.5 ± 12.1	100.7 ± 6.1
*n*-6	83.3 ± 4.9	60.6 ± 9.7	66.8 ± 8.1	83.5 ± 5.8
*n*-6/*n*-3	0.8 ± 0.0	0.9 ± 0.0	0.8 ± 0.0	0.8 ± 0.0
SFA	65.7 ± 9.9	43.7 ± 6.9	43.8 ± 3.5	56.8 ± 2.8
MUFA	36.2 ± 2.4	25.5 ± 4.1	28.5 ± 2.7	37.2 ± 2.9
PUFA	230.5 ± 14.3	156.0 ± 25.0	182.8 ± 22.8	221.4 ± 14.8
AI	0.2 ± 0.0	0.2 ± 0.0	0.2 ± 0.0	0.2 ± 0.0
TI	0.2 ± 0.0	0.2 ± 0.0	0.1 ± 0.0	0.2 ± 0.0
h/H	4.3 ± 0.4	4.3 ± 0.1	4.7 ± 0.2	4.6 ± 0.0

Abbreviations: SFA: saturated fatty acids; MUFA: monounsaturated fatty acids; PUFA: polyunsaturated fatty acids; AI: atherogenicity index; TI: thrombogenicity index; h/H: hypo/hypercholesterolemic ratio.

**Table 2:** Fatty acid profiles and lipid quality indices of *Tetraselmis chui* exposed to different storages conditions (control, light, frozen, and heated), determined by gas chromatography–mass spectrometry (GC–MS) and expressed in μg mg^−1^ of lipid extract. Values are presented as average of three replicates (*n* = 3) ± standard deviation. The fatty acids 16:1 and 18:1 correspond to the sum of two peaks for which the exact position of unsaturation within the chain have not been clearly assigned.

Fatty Acids	Control	Light	Frozen	Heated
16:0	67.6 ± 9.6	54.4 ± 5.6	51.9 ± 5.4	53.0 ± 3.6
16:1	6.6 ± 0.8	6.4 ± 0.8	7.1 ± 0.8	7.1 ± 0.5
16:3*n*-3	3.8 ± 0.4	3.7 ± 0.5	4.3 ± 0.6	4.4 ± 0.3
16:4*n*-3	37.0 ± 4.8	35.3 ± 4.6	41.5 ± 4.5	40.9 ± 4.1
18:0	21.5 ± 5.7	5.0 ± 2.2	3.8 ± 0.3	4.8 ± 1.2
18:1	35.6 ± 3.7	34.1 ± 4.2	35.6 ±3.5	37.8 ± 2.2
18:2*n*-6	18.9 ± 2.1	16.8 ± 2.1	19.3 ± 2.0	20.0 ± 1.3
18:3*n*-6	9.0 ± 1.1	8.3 ± 1.1	10 ± 1.1	9.8 ± 0.9
18:3*n*-3	47.5 ± 4.8	43.7 ± 3.8	51.1 ± 8.4	51.6 ± 6.6
18:4*n*-3	13.9 ± 1.6	13.4 ± 1.4	15.9 ± 2.7	16.1 ± 2.6
20:5*n*-3	13.4 ± 1.5	12.2 ± 1.3	16.0 ± 2.8	15.6 ± 2.2
*n*-3	115.6 ± 11.9	108.3 ± 11.5	128.8 ± 26.7	128.5 ± 15.8
*n*-6	27.9 ± 3.1	25.1 ± 3.2	29.2 ± 4.4	29.9 ± 2.2
*n*-6/*n*-3	0.2 ± 0.0	0.2 ± 0.0	0.2 ± 0.0	0.2 ± 0.0
SFA	89.1 ± 15.3	59.5 ± 7.3	55.7 ± 8.1	57.8 ± 3.0
MUFA	42.3 ± 4.5	40.5 ± 5.1	42.7 ± 6.2	44.9 ± 2.7
PUFA	185.8 ± 19.5	173.9 ± 19.8	200.7 ± 37.3	203.3 ± 20.7
AI	0.4 ± 0.0	0.3 ± 0.0	0.3 ± 0.0	0.3 ± 0.0
TI	0.2 ± 0.0	0.2 ± 0.0	0.1 ± 0.0	0.1 ± 0.0
h/H	2.7 ± 0.2	3.1 ± 0.0	3.7 ± 0.1	3.7 ± 0.1

Abbreviations: SFA: saturated fatty acids; MUFA: monounsaturated fatty acids; PUFA: polyunsaturated fatty acids; AI: atherogenicity index; TI; thrombogenicity index; h/H; hypo/hypercholesterolemic ratio.

**Table 3:** Ratio of non-oxidized and oxidized phosphatidylcholine (PC) and phosphatidylethanolamine (PE) lipid species identified in *Chlorella vulgaris* exposed to different storage conditions (control, light, frozen, and heated). Values are means of the normalized extracted ion chromatograms (XIC) areas of three replicates (*n* = 3) ± standard deviation.

Lipid Species Ratio	Control	Light	Frozen	Heated
PC 34:2/PC 34:2;O	70.6 ± 2.6	26.8 ± 1.3	48.4 ± 16.7	72.2 ± 17.4
PC 34:3/PC 34:3;O	84.2 ± 3.6	41.0 ± 2.9	84.9 ± 5.2	78.7 ± 6.7
PC 34:5/PC 34:5;O	30.7 ± 6.1	23.8 ± 0.5	43.9 ± 1.7	45.5 ± 2.8
PC 36:4/PC 36:4;O	61.3 ± 2.0	40.9 ± 2.4	63.5 ± 7.0	64.0 ± 3.4
PC 36:5/PC 36:5;O	56.9 ± 10.3	38.3 ± 8.2	64.9 ± 2.7	41.4 ± 0.8
PC 36:6/PC 36:6;O	36.0 ± 4.9	29.2 ± 6.8	50.6 ± 2.2	56.0 ± 2.0
PE 32:1/PE 32:1;O	809.3 ± 5.2	23.0 ± 2.9	822.6 ± 65.6	685.4 ± 187.3
PE 34:2/PE 34:2;O	89.8 ± 2.5	36.5 ± 2.5	77.6 ± 4.0	116 ± 46.8
PE 34:3/PE 34:3;O	58.3 ± 5.6	33.4 ± 2.7	48.1 ± 2.7	105.0 ± 9.7
PE 36:3/PE 36:3;O	11.1 ± 0.3	6.5 ± 0.6	11.2 ± 0.7	5.1 ± 0.2

**Table 4:** Inhibition concentration (IC) of lipid extracts (μg mL^−1^) providing 40% and 20% of inhibition (IC_40_, IC_20_) of ABTS^●+^ and DPPH^●^ radicals, respectively, after 120 min of reaction, and the corresponding Trolox-equivalent units (TE) (μmol of Trolox g^−1^ lipid) in samples of *Chlorella vulgaris* exposed to different storage conditions (control, light, frozen, and heated). Results are averages of three assays (*n* = 3) ± standard deviation.

	Control	Light	Frozen	Heated
ABTS				
IC40	32.0 ± 11.0	104.9 ± 6.2	91.1 ± 15.3	49.4 ± 7.0
TE	402.8 ± 173.4	111.1 ± 6.3	131.7 ± 24.1	238.1 ± 31.3
DPPH				
IC20	97.8 ± 27.2	187.9 ± 15.0	103.6 ± 24.6	132.1 ± 2.9
TE	107.1 ± 35.5	52.6 ± 4.2	98.3 ± 21.0	74.5 ± 1.6

**Table 5:** Inhibitory concentration (IC) of lipid extracts (μg mL^−1^) providing 40% and 20% of inhibition (IC4_0_, IC_20_) of ABTS^●+^ and DPPH^●^ radicals, respectively, after 120 min of reaction, and the corresponding Trolox equivalent units (TE) (μmol of Trolox g^−1^ lipid) in samples of *Tetraselmis chui* exposed to different conditions (control, light, frozen, and heated). Results are averages of three assays (*n* = 3) ± standard deviation.

	Control	Light	Frozen	Heated
ABTS				
IC40	41.4 ± 1.0	41.1 ± 0.6	29.0 ± 5.4	88.5 ± 2.8
TE	281.0 ± 6.8	283.0 ± 3.9	411.1 ± 85.2	131.4 ± 4.3
DPPH				
IC20	70.6 ± 23.1	109.4 ± 10.7	90.5 ± 7.0	209.4 ± 10.9
TE	152.5 ± 58.3	90.9 ± 8.4	109.6 ± 8.1	47.3 ± 2.4

## Data Availability

Dataset available on request from the authors.

## Supplementary Materials

The following supporting information can be downloaded at: https://www.mdpi.com/article/10.3390/md22060254/s1, Figure S1:Total ion chromatograms of lipid extract of Chlorella vulgaris under control condition in positive (A) and negative (B) ionization modes by C18-LC-MS; Figure S2: Total ion chromatograms of lipid extract of Tetraselmis chui under control condition in positive (A) and negative (B) ionization modes by C18-LC-MS; Figure S3: LC-MS/MS spectrum in negative mode of PE 32:1 specie at m/z 688.5 corresponding to PE 16:0; 16:1 identified in Chlorella vulgaris under control condition (A) and PE 32:1;O at m/z 704.5 corresponding to PE 16:0; 16:1; O identified in Chlorella vulgaris under light condition (B); Figure S4: GC-MS chromatogram of Chlorella vulgaris under control condition highlighting the identified esterified fatty acids; Figure S5: GC-MS chromatogram of Tetraselmis chui under control condition highlighting the identified esterified fatty acids.
