# Supplementary material for: Tracing the Impact of Domestic Storage Conditions on Antioxidant Activity and Lipid Profiles in the Edible Microalgae Chlorella vulgaris and Tetraselmis chui
Source: Mar Drugs. 2024 May 30;22(6):254. doi: 10.3390/md22060254 (PMC11205134; doi:10.3390/md22060254)
Supplement: Supplementary file 1 [file marinedrugs-22-00254-s001.zip › marinedrugs-3011823-supplementary.pdf]

Tracing the impact of domestic storage conditions on antioxidant activity and lipid profiles in the edible microalgae *Chlorella vulgaris* and *Tetraselmis chui*

Diana Lopes[1,2*], Felisa Rey[1,2], Alexandrina Gomes[2], Luís Duarte[2], João Pereira[1], Marisa Pinho[1], Tânia Melo[1,2], Rosário Domingues[1,2]

[1] Centre of Environmental and Marine Studies (CESAM), Department of Chemistry, Campus Universitário de Santiago, University of Aveiro, 3810-193 Aveiro, Portugal
[2] Mass Spectrometry Centre & LAQV-REQUIMTE, Department of Chemistry, Campus Universitário de Santiago, University of Aveiro, 3810-193 Aveiro, Portugal

*Correspondence: dianasalzedaslopes@ua.pt

Supplementary information

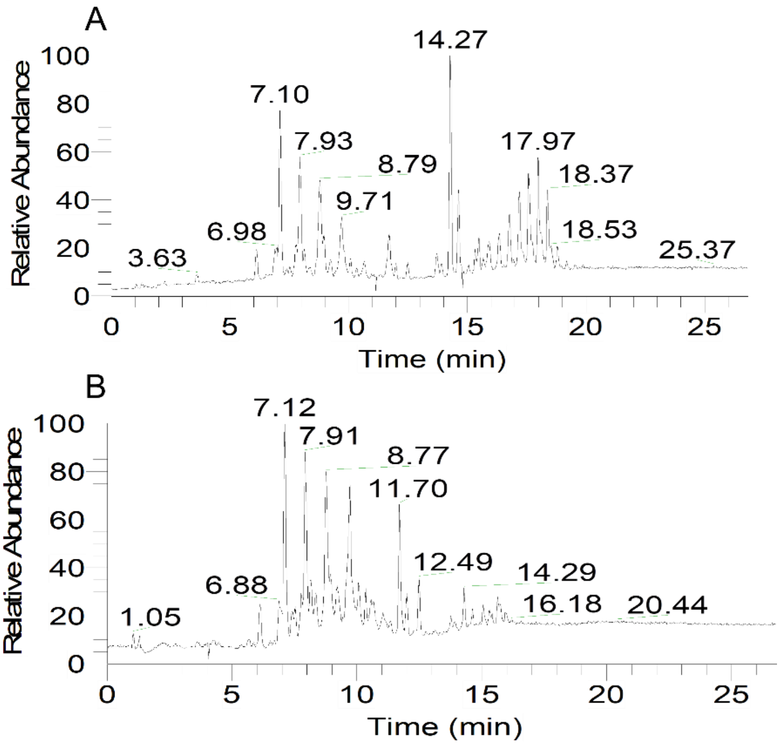

Figure S1. Total ion chromatograms of lipid extract of *Chlorella vulgaris* under control condition in positive (**A**) and negative (**B**) ionization modes by C18-LC-MS

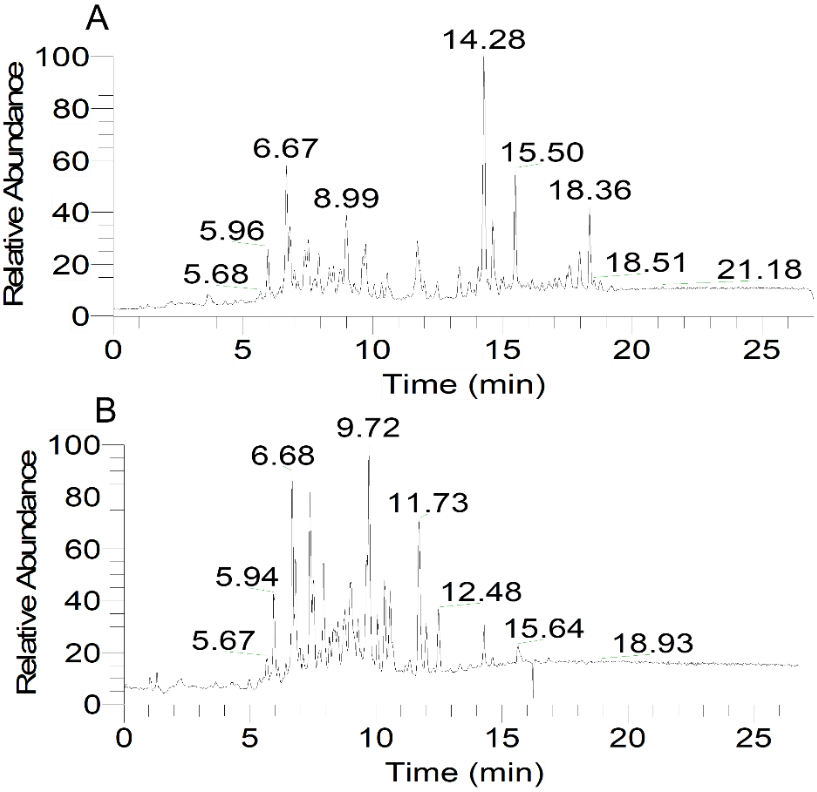

Figure S2. Total ion chromatograms of lipid extract of *Tetraselmis chui* under control condition in positive (**A**) and negative (**B**) ionization modes by C18-LC-MS

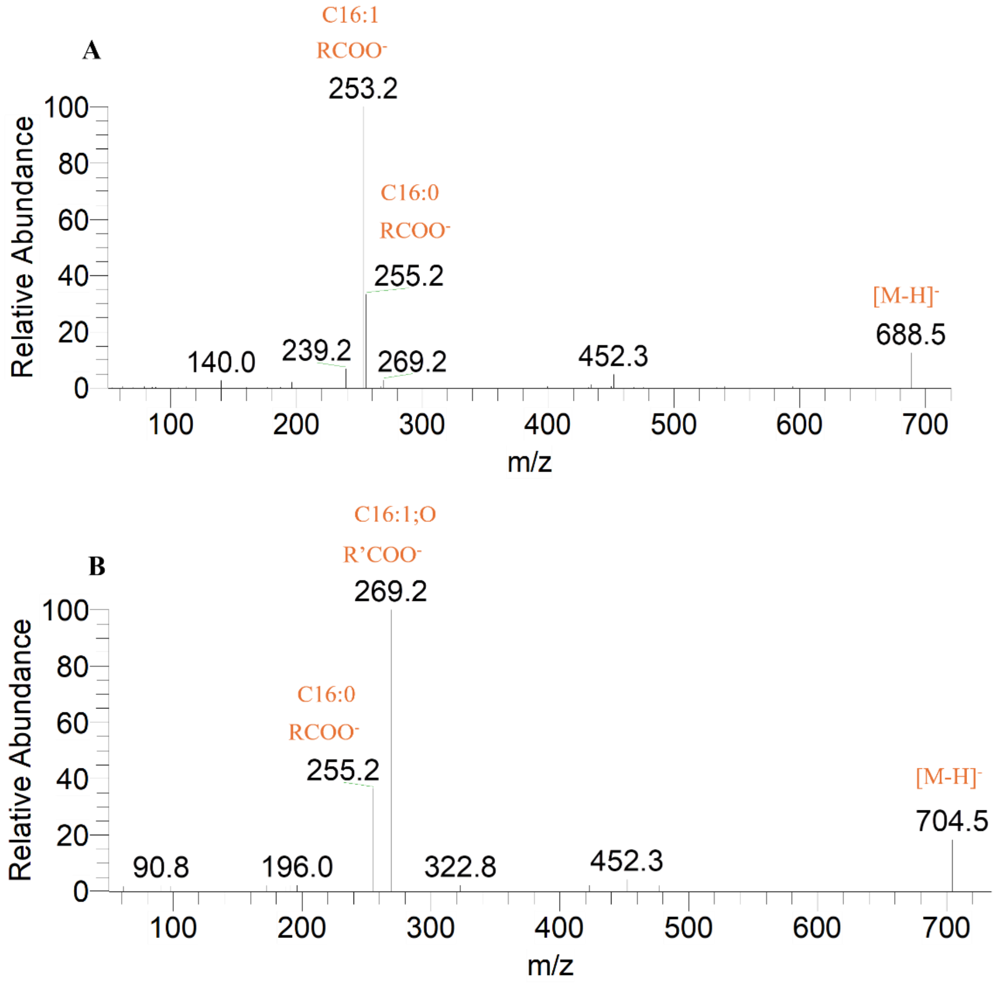

Figure S3. LC-MS/MS spectrum in negative mode of PE 32:1 specie at m/z 688.5 corresponding to PE 16:0; 16:1 identified in *Chlorella vulgaris* under control condition (**A**) and PE 32:1;O at m/z 704.5 corresponding to PE 16:0; 16:1; O identified in *Chlorella vulgaris* under light condition (**B**).

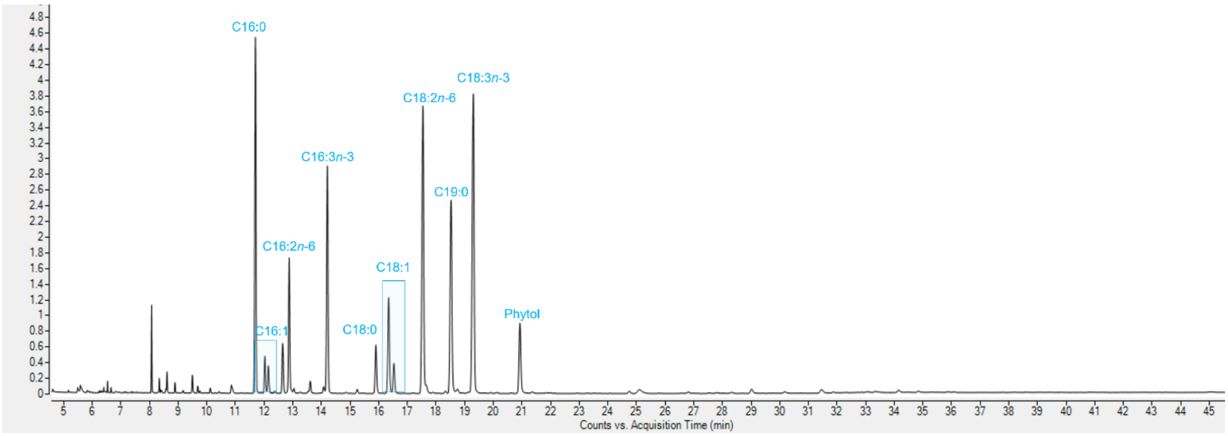

Figure S4. GC-MS chromatogram of *Chlorella vulgaris* under control condition highlighting the identified esterified fatty acids.

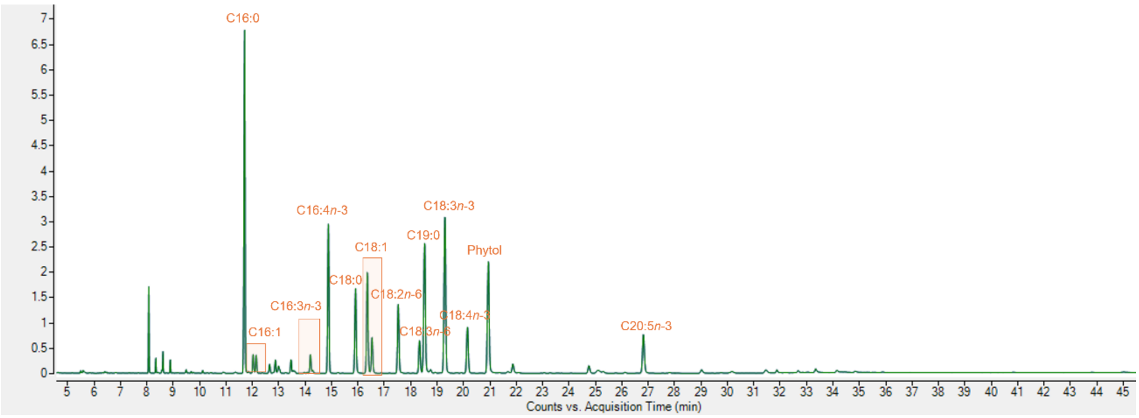

Figure S5. GC-MS chromatogram of *Tetraselmis chui* under control condition highlighting the identified esterified fatty acids.
